# Impact of Nursing Methodology Training Sessions on Completion of the Virginia Henderson Assessment Record

**DOI:** 10.3390/nursrep10020014

**Published:** 2020-11-25

**Authors:** Maria Lopez, Jose-Maria Jimenez, Mercedes Fernández-Castro, Belen Martin-Gil, Sara Garcia, Maria-Jose Cao, Manuel Frutos-Martin, Maria-Jose Castro

**Affiliations:** 1 Nursing Faculty, University of Valladolid, Avenida Ramon y Cajal 5, 47005 Valladolid, Spain; maria.lopez.vallecillo@uva.es (M.L.); sara.garcia.villanueva@uva.es (S.G.); mjcao@enf.uva.es (M.-J.C.); mafruel@enf.uva.es (M.F.-M.); mariajose.castro@uva.es (M.-J.C.); 2 Hospital Clínico Universitario de Valladolid, Av. Ramón y Cajal, 3, 47003 Valladolid, Spain; mefernandezc@saludcastillayleon.es (M.F.-C.); bmartingi@saludcastillayleon.es (B.M.-G.)

**Keywords:** electronic health records, needs assessment, education, nursing care

## Abstract

The Virginia Henderson model, integrated in the computer application GACELA Care, helps to standardise the nursing assessment and establish precise and personalised nursing diagnoses. The aim was to determine the extent of completion of the initial patient assessment record after nurses following a training programme on nursing methodology. A quasi-experimental, retrospective, randomised, observational, single-group study was performed in two stages: pre-training and post-training. Voluntary training sessions were held for the nurses that work with GACELA Care. The completion of the initial patient assessment using the needs of Virginia Henderson and the Norton scale was evaluated before and after the training sessions. Completion of the needs of Virginia Henderson in the initial patient assessment increased from 94.2% to 100% (*p* = 0.014). Completion of “hygiene/skin” increased significantly from 83.3% to 95.8% (pre-training and post-training, respectively). The remaining needs did not show statistical significance. Recording of the Norton scale increased from 63.13% to 92.5% (*p* < 0.001). The training sessions on nursing methodology have improved the completion of records and inclusion of normal characteristics, defining characteristics and risk factors, and improving pressure ulcer risk assessment through the Norton scale.

## 1. Introduction

Correct application of the nursing process is understood as “an action full of meaning” that constitutes a professional challenge “in education and in practice” [1]. Ongoing professional training is directly involved in the completion of nursing records by means of encouraging reflective practice that allows work to be performed with scientific evidence applied to healthcare [2].

Effective care guidance requires systematic and rational planning through an appropriate method, using the theoretical framework as a tool to aid recording of nursing activity and to reinforce this activity “as a discipline, science and profession that bases its practice on theories and on philosophical and ethical principles” [3]. Teresa Luis Rodrigo explained the need to work with a nursing model that can be used to specify the “nature of care” and that helps to “define the area of nursing competency”. One option is to use Virginia Henderson’s needs theory, one of the most well-known nursing models. Henderson’s terminology is directly related to nursing interventions and the attainment of nursing objectives. By carrying out a reliable and comprehensive assessment based on observation and interviewing, nurses can establish precise and personalised nursing diagnoses as part of their care plan. It is considered crucial to record this care plan, as it facilitates communication among professionals, acts as a guide for implementing care and enables evaluation of the work performed [4]. Documenting nursing care through records is an important part of professional activity, not only for ethical and legal reasons, but also because there is a need to reflect care practice and heighten the visibility of the profession. The evaluation of these records encourages health professionals to complete them appropriately, concisely and thoroughly, and contributes to increased quality of records and quality of care [5]. Nursing records form part of patients’ clinical history, as they constitute “assessments and information of any nature about patients’ situation and clinical progress throughout their care process, the correct completion which is the responsibility of the health professional” [6]. Switching to digital format will alter the recording method but not the contents of the documents. This means electronic clinical records will continue to act as the hub of patients’ clinical information and the support for communication among the different health professionals treating the patients [7].

Despite the importance of working with nursing methodology [8], the implantation and systematic organisation of the five phases of the process—assessment, analysis, diagnosis, implementation and evaluation—is no simple task [9]. Computerisation of clinical records can help to make this process more efficient. The American Nurses Association asserts that electronic nursing records must promote and facilitate decision-making and communication, and support critical thinking by storing accurate and efficient information [10].

At the Hospital Clínico Univeristario de Valladolid (HCUV) the online nursing care management computer programme GACELA Care (GACELA being the acronym for the programme title in Spanish), Version 1.8-09, enables the nursing staff to integrate nursing methodology into their daily work. The application makes it easier to complete the progress notes, clinical variables, nursing care plan and discharge care report contained in patients’ clinical records in accordance with legal provisions. GACELA Care therefore tracks the assessment, planning and implementation of the nursing care provided to the hospitalised patient [6].

In the HCUV, as a first step in the continuous care process for admitted patients, a nursing assessment is performed using the needs of Virginia Henderson. Through this model, the interview is effectively structured and the record of the interview is standardised through the NANDA-I nursing diagnoses taxonomy. As part of protocol, admitted patients are also assessed for risk of pressure ulcer (PU) development using the Norton scale. In this way, specific nursing interventions can be included in the care plan to prevent PU development [11].

There is a direct link between staff training and improvement in the documentation collected. In some studies, implementing pain assessment training programmes improved management and recording of pain [12]. Training sessions aimed at improving quality of nursing records are deemed necessary because, according to López et al., nursing staff do not have the knowledge or tools needed to create high-quality clinical records that would help improve patient care [13].

The idea of offering training sessions was put forward with the aim of improving the recording of nursing assessment data in the HCUV. The sessions would act as a point of contact for the nurses and would enable them to update and improve their knowledge and skills [14]. The programme was included in the hospital’s Annual Continuous Training Plan (2013, 2014, and 2015). The objectives, content, recipients, trainers and support systems were designed and planned in accordance with the established quality criteria. The sessions were designed to help nurses update their knowledge, skills and abilities, in order to achieve greater professional development and improved quality of care [11]. Training must be aimed at changing workers’ behaviour in order to improve organisational efficacy, since learning constitutes a reorganisation of acquired knowledge [15].

On the basis of these criteria, the training sessions were implemented with a view to improving completion of the Virginia Henderson needs assessment and the PU risk assessment. The sessions took into account the competency profile of the nurses, were tailored to practical application and made use of new technologies.

Chapter XII of Spanish law 2/2007 of 7 March on the legal status of statutory health service staff in Castilla y León states that “The results of planned training must be evaluated, not only through the knowledge acquired, but also by assessing the impact on professional performance and quality of services” [16]. For this reason, the extent of completion of the initial patient assessment and the Norton scale in the GACELA Care application were to be evaluated after the training sessions had taken place.

The purpose of this study was to analyse the contribution of a nursing methodology training programme to the degree of completion of the patient’s assessment.

## 2. Materials and Methods

### 2.1. Study Design

A quasi-experimental, single-group study was performed in two stages: pre-training and post-training.

### 2.2. Participants

The study population comprised 197 nurses who worked in the medical and surgical inpatient units of the HCUV and who recorded the care provided in the GACELA Care application. The nurses’ participation in the training sessions was voluntary. In the last quarter of 2013, 2014 and 2015, the sessions were attended by 127, 142 and 139 nurses, respectively. (Approved by HCUV Research Committee, CINV 16-94).

### 2.3. Data Collection

Completion of the initial patient assessment on admission and the Norton scale in the GACELA Care application was evaluated in a cross-section performed in June 2013. Training sessions were then held in the last quarter of 2013, 2014 and 2015. After the sessions had taken place, completion of the initial patient assessment and Norton scale was evaluated again in April 2016.

### 2.4. Study Sample

The study sample comprised the records of the initial patient assessment on admission and the Norton scale assessment. The records were divided into two groups: group 1 comprised 120 assessment records completed between 1 and 30 June 2013; group 2 comprised 120 assessment records completed between 1 and 30 April 2016, after the training sessions included in the Annual Training Plan had taken place. The sample size was calculated accepting an alpha risk of 0.05 and a beta risk of 0.2 in a two-sided test. This calculation showed that 120 subjects were needed, assuming an initial compliance rate of 40% and final compliance rate of 60%. The records were selected through systematic random sampling. Only records that followed the Virginia Henderson model in adult patients admitted for more than 24 h in the inpatient units of the HCUV met the inclusion criteria.

The variables studied were 11 of the 14 basic human needs identified by Virginia Henderson: “breathe normally”, “eat and drink”, “eliminate body wastes” “movement”, “sleep and rest”, “dress and undress”, “temperature”, hygiene/skin”, “safety” and “communicate and learn”. These needs were chosen as they are mandatory completion fields while the needs “values and beliefs”, “work/accomplishment” and “recreational activities” are optional. The remaining variables analysed were: 52 normal characteristics (NC); 79 defining characteristics (DC); 43 risk factors (RF); the completion or otherwise of the Norton scale, and demographic data such as age, gender and medical diagnosis.

### 2.5. Training Sessions

The sessions were voluntary and classroom-based, and included both theoretical and practical aspects. They were taught in the HCUV computer room by two nurses who are responsible for the GACELA Care application in the HCUV. The sessions lasted 30 min and were held from Monday to Friday over one month, in the last quarter of 2013, 2014 and 2015. They were held at three different times each morning to facilitate attendance and resolution of doubts. All the sessions were accredited by the institution with a diploma certifying attendance.

The theoretical content of the sessions was centred on the methodology of nursing care, the initial patient assessment and the Norton scale, using the standardised language NANDA-I, NOC and NIC, and based on the literature and the documentation prepared in the HCUV. The practical block began with a practical demonstration of how to complete the records. Each participant then carried out a practical case individually on a computer, using the training version of the GACELA Care application.

### 2.6. Ethical Considerations

Before carrying out the proposed study, written permission and approval was obtained from the HCUV Research Committee, with the internal code CINV 16-94. The confidentiality of the data provided in the initial patient assessment was maintained. Informed written consent was obtained from all subjects. The study adhered to the Declaration of Helsinki (2013).

### 2.7. Statistical Analysis

The data were collected through the database management system ACCESS (Microsoft Office. 2007). All data were processed anonymously. They were entered by one person and reviewed by an expert.

The software IBM SPSS Statistics Version 20.0 was used to carry out the statistical analysis. The quantitative variables were expressed as mean and standard deviation and the qualitative variables as frequencies and percentages. The pre-training and post-training groups were compared using Pearson’s chi-squared test. The level of statistical significance was set at *p* < 0.05.

## 3. Results

Completion of the record of the initial patient assessment on admission was evaluated. There were no significant differences between the groups in the variables gender, age and medical diagnosis. Group 1 (120 records, 2013) had a mean age of 66.71 ± 18.47 years. Of all the group 1 records, 54.2% concerned men and 45.8% concerned women. Group 2 (120 records, 2016) had a mean age of 64.40 ± 18.62 years. As with group 1, there were more male than female patients (52.5% vs. 47.5%). With regard to type of medical diagnosis, records of patients admitted to the Internal Medicine department predominated in both groups (25.8%).

Completion of the 11 needs of Virginia Henderson in the initial patient assessment increased from 94.2% to 100% (*p* = 0.014). Of the 11 needs, completion of “hygiene/skin” showed the largest statistical significance between the pre-training and post-training groups, increasing from 83.3% to 95.8% (*p* = 0.002). The remaining needs did not show statistical significance (Table 1).

Of all the DCs, RFs and NCs included in the Virginia Henderson assessment, 7 DCs, 12 RFs and 8 NCs related to the needs “breathe normally”, “eat and drink”, “eliminate body wastes”, “hygiene/skin”, “safety” and “communication” were selected 0 times in both G1 and G2. (Table 2).

The following NCs were chosen on more than 30% of occasions, in both G1 and G2: “walks without help”, “sufficient restorative sleep”, “dresses and undresses alone” “temperature within normal limits” “autonomy in self-care (hygiene)” and “appears alert and oriented” (Table 3).

As regards DCs and RFs, only the DC “nervousness” included in the “safety” need showed statistical significance (*p* = 0.010), increasing from 6.7% to 17.5%. Recording of the Norton scale increased from 63.13% to 92.5% (*p* < 0.001).

## 4. Discussion

Our study analysed the changes in completion of the initial patient assessment record following a training programme on nursing methodology in the HCUV. The data obtained show an improvement in completion of the assessment post-training. As in other studies, training has been shown to have a positive effect on the nurses’ skills [17,18,19].

The training activity did not affect the completion of demographic data, since the variables gender, age and medical diagnosis appear automatically in the record. Demographic data were collected in order to analyse how they related to the extent of record completion. Most of the records covered a population aged over 65 years.

Completion of the initial patient assessment record in the HCUV increased after the training activities on nursing methodology had taken place, reaching a rate of 100%. A significant improvement was also achieved in the assessment of pressure ulcer risk with the Norton scale, indicating that the training sessions had been effective. Other authors have demonstrated a direct link between training of nurses and recording of nursing activities [13,15]. Evaluating completion of the Norton scale is one of the objectives set in the Castilla y León Health Care System Annual Management Plan (SACYL), as this tool is an indicator of nursing quality and it directly affects the nurses working in the HCUV. Literature reviews and good clinical practice guides stress the importance of assessing the skin of all patients who are admitted to hospital [11].

In our study, the assessment of the “hygiene/skin” factor was the only one that improved significantly after the implementation of the training programme. This need is directly involved in the assessment of the state of the skin, the degree of patient autonomy in personal hygiene, and as one of the elements that allows the identification of patients with PU risk. It may be useful to carry out further studies to determine why some DCs, NCs and RFs were not selected in any of the assessments analysed. Some authors have shown that, inter alia, the complexity of evaluating certain needs, such as “values and beliefs”, “communication” and “safety”, can hinder assessment completion [9]). Several other barriers to assessment have been identified, including: lack of patient collaboration; lack of protocols; constant repetition of tasks over the working day; clinical processes that require the collaboration of the family member, who is absent at the time of data collection; lack of initiative of professionals with regard to continuous training; and the fact that some staff do not believe the assessment has any practical use. Advocates of research and monitoring of care processes to evaluate nursing intervention recommend that care be computerised and standardised [20]. This computer record helps to standardise care, keeps track of the work performed and eases communication and continuity of care. The problem is that this computerisation is seen by nurses as a further increase in workload [21].

Many authors assert that nurses must be involved in the analysis and preparation of the documents that are used to decide which information will be useful [22]. They stress that nurses themselves “need to decide what data should be included in the electronic record”, and what terminology adequately reflects nursing interventions and results [21]). This is crucial as the absence of nursing records constitutes ethical and professional misconduct, which raises doubts as to whether the nursing professional is assuming responsibility for his or her interventions [23].

It should be noted that the NCs chosen most frequently were the same in both groups and were not affected by the training sessions. All share the common trait of being the first to appear in the drop-down menu of the application. The option “temperature within normal limits” is the only available NC in the need “temperature”, and only two NCs appear for the needs “rest/sleep” and “dress and undress”.

In this study, the DC “nervousness”, included in the need “safety”, was selected more frequently post-training, despite other authors showing that this need is one of the most difficult to assess [9]. Building on knowledge in the areas of the Virginia Henderson model that are difficult to assess may therefore facilitate assessment [11].

Working with the Virginia Henderson assessment in GACELA Care leads to standardisation of care, thus helping to enhance the professional role of nurses and reinforce the use of international nursing language [24]. This computer tool guides clinical decision-making, makes it easier to document the nursing process and improves care practice. Carrying out the assessment in GACELA care enhances the visibility of nursing, makes it easier to evaluate the impact of care and helps to improve continuity of care. The fact that this software is adapted to nursing methodology improves the accuracy and precision of the recorded data, making them easier to measure [25].

Training nurses with formative programmes has proven to be a positive strategy to achieve better record keeping. However, there are a number of barriers, such as lack of time, healthcare demands, lack of institutional support, etc., to encourage the development of skills based on training programmes for nursing staff [26].

One of the most important implications of our study for clinical practice is the increase in nursing skills related to completion of the initial patient assessment record as a result of continuous training. Scheduling continuous training programs on nursing methodology strengthens the link between nursing assessment and care planning, which is reflected in the quality of care provided to the patient. A possible future line of research could be measuring the link between the quality of the completed nursing records and the quality of care provided.

The results of the present study show a positive correlation between continued training and improvement in patient assessment. This will translate into precise nursing diagnoses that will lead to nursing interventions tailored to the needs of each patient.

One important limitation of this study is the fact that the nursing assessment on admission is subject to the variability of the patient’s clinical status. Choosing medical and surgical inpatient units increased the diversity of the types of patients assessed, resulting in heterogeneity in the needs assessed and care planning. The choice of NCs, DCs and RFs were therefore conditioned by the patient’s status and degree of autonomy. The second limitation is in regard to attendance of the training sessions. While every effort was made to ensure the sessions were accessible to staff, participation was voluntary, and it was therefore not possible to include 100% of the nurses working in the inpatient units. This means that some of the records evaluated in our study may have been completed by nurses who did not attend the training sessions.

## 5. Conclusions

The training sessions on nursing methodology have had a positive impact on initial patient assessment, improving completion of the records and inclusion of NCs, CDs and RFs, and improving pressure ulcer risk assessment through the Norton scale. Improved completion of the initial patient assessment makes it easier to develop a care plan using the DCs and RFs selected.

This study reveals the importance of performing the initial assessment of hospitalized patients in a coordinated fashion in all the hospitals of the Spanish National Health System, and promoting joint training strategies in an effort to develop the skills and specific capabilities of nursing professionals. The initial training sessions should also be followed up with knowledge updates at different time intervals in order to develop good nursing practice and thus improve quality of patient care.

## Figures and Tables

**Table 1:** Completion of nursing assessment.

Needs of Virginia Henderson	Pre-Training		Post-Training		*p*-Value
	%	*n*	%	*n*	
Breathing	68.3	82	72.5	87	0.479
Eat/Drink	65.8	79	72.5	87	0.263
Eliminate body wastes	74.2	89	75.8	91	0.766
Movement	72.5	87	73.3	88	0.885
Rest/sleep	64.2	77	68.3	82	0.495
Dress and undress	68.3	82	66.7	80	0.783
Temperature	61.7	74	66.7	80	0.419
Hygiene/skin	83.3	100	95.8	115	0.002
Safety	76.7	92	82.5	99	0.562
Communication	35.8	43	35.8	43	1
Learn	47.5	57	42.5	51	0.436

**Table 2:** Normal characteristics, defining characteristics and risk factors not selected.

Need	Normal Characteristic	Defining Characteristic	Risk Factor
Breathe normally		Nasal flaring	Facial surgery or trauma
			Delayed gastric emptying
Eat and drink	Esophagostomy: working normally	Thirst	
	Gastrostomy: working normally		
	Duodenostomy: working normally		
	Jejunostomy: working normally		
Eliminate body wastes	Ileostomy: working normally	Unable to pass stool	Laxative overdose
	Regular menstruation		Rectal stenosis
	Last Menstrual Period LMP		Pregnancy
Hygiene/skin		Xerostomia (dry mouth)	Circulatory disorder
		Stomatisis	
		Bleeding	Radiation
Safety		Shaking/trembling hands	Immunosuppression
			History of previous injuries
			Reduced thermal or tactile sensitivity
			Overexposure to the sun, sunlamps, radiation therapy
			History of previous suicide attempts
Communication	Hearing aid well fitted		

**Table 3:** Most frequently selected normal characteristics in the initial patient assessment.

	Pre-Training		Post-Training	
	%	*n*	%	*n*
Non-smoker	15.8	19	19.2	23
Pink skin and mucous membranes	21.7	26	24.2	29
Normal breathing rhythm and rate	20.8	25	22.5	27
Autonomy in self-care	33.3	40	40	48
Chews without difficulty	23.3	28	25.8	31
Swallows without difficulty	15	18	25.8	31
Urinary continence	15	18	20	24
Bowel continence	15	18	16.7	20
Insufficient physical activity	18.3	22	24.2	29
Limited scope of movement	25	30	25.8	31
Walks without help	34.2	41	34.2	41
Verbal complaints of not feeling well rested	11.7	15	12.5	15
Sufficient restorative sleep	31.7	38	36.7	44
Dresses and undresses alone	39.2	47	34.2	41
Temperature within normal limits	55	66	55	66
Autonomy in self-care (hygiene)	31.7	38	30	36
Skin intact	27.5	33	21.7	26
Appears alert and oriented, and responds appropriately	40	48	48.3	58
Phonation, vocal function, register and tone maintained	10.8	13	16.7	20
Knows about his/her disease	28.3	34	27.5	33
Appears able and willing to learn	21.7	26	20.8	25
